# Personality stability and change across the academic semester

**DOI:** 10.3389/fpsyg.2025.1531794

**Published:** 2025-06-27

**Authors:** Stephanie M. Anglin, Rachel S. Rubinstein, Dustin A. Haraden, Caitlin Drummond Otten, Brandon Mangracina, K. Mackenzie Shaw

**Affiliations:** ^1^Department of Psychological Science, Hobart and William Smith Colleges, Geneva, NY, United States; ^2^Department of Psychology, Towson University, Towson, MD, United States; ^3^Department of Psychology, Rochester Institute of Technology, Rochester, NY, United States; ^4^School of Human Evolution and Social Change, Arizona State University, Tempe, AZ, United States

**Keywords:** personality stability, personality change, personality development, situation, Five-Factor model, personality processes, college students, young adulthood

## Abstract

This study tested competing hypotheses of student personality change across the academic semester, and examined the academic, social, extracurricular, health, and affective experiences associated with changes. Previous research suggests that personality can vary substantially in response to situational factors (Situational Perspective) but shows high levels of consistency over time (Personality Stability). Despite consistency, research also finds developmental patterns of change, particularly in transitional periods such as college as young adults adapt to social role changes (Maturity Principle). We asked college students to complete measures of personality and experiences at the beginning and two-thirds of the way through the Fall semester. The Situational Perspective predicts that personality will change in response to changes across the semester (e.g., in workload), with conscientiousness and extraversion decreasing and neuroticism increasing, while the Maturity Principle predicts that conscientiousness and agreeableness will increase and neuroticism will decrease as students adapt to new roles and expectations, and the Personality Stability Perspective predicts that personality will remain unchanged. We found a decrease in conscientiousness, consistent with the Situational Perspective, along with decreases in agreeableness and openness, which were unpredicted from all three theories. Changes in personality co-occurred with declines in subjective wellbeing, social support, and health behaviors. Our results extend prior research observing personality changes associated with maturity over the college years, finding short-term declines in traits associated with maturity over the semester. Although further research is needed, these findings may suggest that college students must face and adapt to new challenges and expectations before growing from their experiences.

## 1 Introduction

Studies of personality change have investigated how personality states vary over short time periods (e.g., hour-to-hour and day-to-day; Fleeson, 2001, 2007; Heller et al., 2007) in response to contextual factors. Research has also examined trends of personality change over long time periods (e.g., years and decades; e.g., Atherton et al., 2021; Caspi et al., 2005; Roberts et al., 2006; Roberts and DelVecchio, 2000), focusing on transitional periods and lifespan developmental patterns of change. Fewer studies have examined personality change in time periods between days and years, including the span of the college semester. We build on prior longitudinal studies of college students, which have assessed personality change over 4 years of college (e.g., Atherton et al., 2021; Klimstra et al., 2018; Roberts and Robins, 2004; Vollrath, 2000) and in the transition from high school to college (Lüdtke et al., 2009), as well as studies that have tested for personality differences between students who participate in research early vs. later in the semester (Aviv et al., 2002; Ebersole et al., 2016; Witt et al., 2011). The present study tracked college students across the semester to test for trends in personality change that may correspond to varying experiences (academic, social, extracurricular, health, and affective) during different periods of the semester. We examine competing hypotheses regarding patterns of personality change over the semester and how personality changes correspond to changes in experiences across the semester. To our knowledge, prior research has not tested these competing theories of personality change across the semester and how changes correspond to experiences across areas of students' lives during this time.

### 1.1 Theories of personality stability and change

#### 1.1.1 Personality stability

From the Personality Stability perspective (or the “plaster hypothesis”; described in McCrae and Costa, 1994), personality traits are stable over time, especially over short time intervals (e.g., Caspi et al., 2005; McCrae and Costa, 1994). According to this perspective, meaningful personality change is only seen over long time periods such as years and decades. Longitudinal studies show rank-order stability of personality traits, with moderate test-retest correlations from childhood to adolescence and increasingly strong correlations from young adulthood onward (Roberts and DelVecchio, 2000; Caspi and Roberts, 2001). Therefore, from the Personality Stability perspective, personality would not change within the semester.

#### 1.1.2 Maturity principle

From a developmental perspective, personality changes in response to social roles (Roberts et al., 2006). Indeed, despite high rank-order stability, personality shows consistent patterns of mean-level change over time (e.g., Atherton et al., 2021; Caspi et al., 2005). Based on the maturity principle of personality development, normative patterns of change occur from adolescence to adulthood as young adults adapt to social role changes. Specifically, conscientiousness and agreeableness increase and neuroticism decreases to meet the expectations and responsibilities of adult roles (McCrae et al., 2000; Roberts et al., 2006; Soto et al., 2011; Srivastava et al., 2003). These changes are often observed during transitional periods (Lüdtke et al., 2009), particularly over the college years (Atherton et al., 2021; Klimstra et al., 2018; Roberts and DelVecchio, 2000; Roberts et al., 2006; Robins et al., 2001, 2005; Vaidya et al., 2002; Vollrath, 2000), and even over the span of a few months (Bleidorn, 2012). Thus, based on the Maturity Principle, personality changes that are consistent with this principle would occur over the semester.

#### 1.1.3 Situational perspective

From a situational perspective, personality varies across contexts. Studies show considerable variation in personality from hour-to-hour and day-to-day in response to situational factors (i.e., personality states; Fleeson, 2001, 2007; Fleeson and Gallagher, 2009; Geukes et al., 2017; Heller et al., 2007). Personality traits are theorized to be the average of personality states over time (Fleeson, 2001; Fleeson and Gallagher, 2009). People perceive their personality differently across situations (e.g., family, work, friends; Nasello et al., 2023), and personality changes in response to context (e.g., Lüdtke et al., 2011). Thus, from a situational perspective, patterns of change in response to varying experiences over the semester may differ from long-term developmental patterns of change. For example, individuals may decrease rather than increase in conscientiousness and increase rather than decrease in neuroticism in the shorter-term (i.e., across the semester) before adapting and changing from these experiences over the college years. Indeed, students who participate in studies early in the semester report higher conscientiousness, more positive mood, and lower stress than those who participate later (Aviv et al., 2002; Ebersole et al., 2016; Witt et al., 2011). Therefore, from the Situational Perspective, personality would change in response to changes in experiences across the semester (e.g., light vs. heavy workload, a lot vs. little free time and stress, etc.), likely in different ways than the maturity principle would predict.

### 1.2 Present study

Although research has not specifically examined personality change across the semester and how changes correspond to experiences during this eventful time period, Bleidorn (2012) assessed personality at the beginning of the first and second semester of the first year of college, observing an increase in conscientiousness and openness and decrease in neuroticism. Researchers have also tested how personality predicts behavior across the semester (e.g., Kroencke et al., 2019) and students' volitional personality change efforts each week of the semester (Hudson et al., 2019). Prior studies have found several variables associated with variability in personality states and personality change, including daily hassles (Vollrath, 2000), subjective wellbeing, positive and negative affect (Heller et al., 2007), contextual and situational cues (Fleeson, 2001; Fleeson and Gallagher, 2009; Geukes et al., 2017), exercise (Kroencke et al., 2019), goals (Roberts et al., 2004), and life events/experiences (e.g., Lüdtke et al., 2011; Vaidya et al., 2002). We build on prior research to examine different possible short-term situational and long-term developmental patterns of change and a broad range of experiences across areas of students' lives that may be associated with these changes. In so doing, this study aimed to advance theory and research on personality change and provide important methodological information for researchers conducting studies at different times of the semester.

This study examined personality stability and change over the semester by measuring personality at the beginning (T1) and two-thirds of the way (T2) through the Fall semester. We chose two-thirds of the way through the semester for T2 as workload would be high but not so high that participants would fail to complete Part 2. Participants completed measures of personality, subjective wellbeing, social support, health behaviors, and academic, extracurricular, work, and social activities at each time point. This design enabled us to test personality consistency and change from T1 to T2, consistency and change in experiences from T1 to T2, how personality and experiences correspond at each time point, and whether changes in personality are associated with changes in experience from T1 to T2. Due to the potential for reciprocal associations between personality and experiences over time, we also conducted exploratory analyses examining how personality and experiences at T1 may predict one another at T2.

#### 1.2.1 Hypotheses

##### 1.2.1.1 H1: Personality change

The theories of personality stability and change described above produce competing predictions about whether and how personality will change over the semester. We provide a summary of these predictions in Table 1.

**Table 1:** Predicted and observed patterns of personality change.

**Trait**	**Five-factor model**	**Maturity principle**	**Situational perspective**	**Observed findings**	**Disruption hypothesis**
Conscientiousness	No change	Increase	Decrease	Decrease	Decrease
Neuroticism	No change	Decrease	Increase	Inconclusive	No prediction
Extraversion	No change	No prediction	Decrease	Inconclusive	No prediction
Openness	No change	No prediction	Increase	Decrease	Decrease
Agreeableness	No change	Increase	No change	Decrease	Decrease

The disruption hypothesis is a post-hoc explanation offered to explain the unexpected findings.

###### 1.2.1.1.1 Conscientiousness

New beginnings are associated with increases in conscientiousness (Leikas and Salmela-Aro, 2015; Lüdtke et al., 2009), those who participate in studies earlier in the semester report higher conscientiousness than those who participate later (Aviv et al., 2002; Ebersole et al., 2016; Witt et al., 2011), and increasing workload over the semester may make it difficult to maintain initial levels of conscientiousness. Therefore, the Situational Perspective (SP) predicts that conscientiousness will decrease from the beginning (T1) to the middle-end (T2) of the semester. Alternatively, because conscientiousness increases across college (Atherton et al., 2021; Klimstra et al., 2018; Robins et al., 2001; Vollrath, 2000), even from the first to second semester (Bleidorn, 2012), the Maturity Principle (MP) predicts that conscientiousness will increase as participants adapt to increasing demands. The Personality Stability Perspective (PSP) predicts no change.

###### 1.2.1.1.2 Neuroticism

The first semester of college is stressful (Bewick et al., 2010; Larcombe et al., 2016; Pitt et al., 2018), stress increases over the semester (Fuller et al., 2003; Pitt et al., 2018), particularly during exam periods (Garett et al., 2017), and participants in studies later in the semester report less positive mood and greater stress (Aviv et al., 2002; Ebersole et al., 2016; Witt et al., 2011). Because neuroticism is associated with stress and responding more negatively to stress (Bolger and Zuckerman, 1995), based on the SP, neuroticism will increase from T1 to T2. However, because neuroticism declines over the college years (e.g., Atherton et al., 2021; Klimstra et al., 2018; Lüdtke et al., 2009; Robins et al., 2001; Vollrath, 2000), even from the first to second semester (Bleidorn, 2012), based on the MP, neuroticism will decrease from T1 to T2 as students acclimate to the new environment. The PSP predicts no change.

###### 1.2.1.1.3 Extraversion

As the beginning of the semester is a time of making and reuniting with friends, enthusiasm about new courses, and a lower academic workload, from the SP, extraversion will decrease from T1 to T2. Of the Big Five traits, extraversion varies the most in the short-term (Fleeson, 2001) but is consistent in the long-term (Atherton et al., 2021; Klimstra et al., 2018; Robins et al., 2001; Roberts and DelVecchio, 2000), and changes little over the college years (Atherton et al., 2021; Robins et al., 2001). Therefore, a competing hypothesis is that extraversion levels will remain unchanged, consistent with the PSP. No direct prediction follows from the MP.

###### 1.2.1.1.4 Openness

College is a time for exploring new opportunities, ideas, and ways of thinking. Some studies have shown that openness increases over the college years (Lüdtke et al., 2009; Robins et al., 2001; Vaidya et al., 2002), even from the first to second semester (Bleidorn, 2012), though changes may be small (Atherton et al., 2021) and have not emerged in all studies (e.g., Vollrath, 2000). Therefore, the SP predicts that openness may increase from T1 to T2, whereas the PSP predicts no change. No direct prediction follows from the MP.

###### 1.2.1.1.5 Agreeableness

Some studies have found that agreeableness increases over the college years (Atherton et al., 2021; Lüdtke et al., 2009; Robins et al., 2001; Vollrath, 2000), consistent with the MP (Caspi et al., 2005). However, others have found mixed results or little change (Klimstra et al., 2018; Vaidya et al., 2002), including from the first to second semester (Bleidorn, 2012). Agreeableness also shows the least variability in personality states (Fleeson, 2001). The MP predicts that agreeableness will increase, whereas the SP and PSP predict no change.

##### 1.2.1.2 H2: Changes in experience

We expect an increase in stress and academic workload (Larcombe et al., 2016; Pitt et al., 2018) and decrease in subjective wellbeing (Aviv et al., 2002; Ebersole et al., 2016; Witt et al., 2011) and health behaviors (e.g., Kroencke et al., 2019) from T1 to T2, based on previous research demonstrating these patterns. We also expect a decline in social activity to co-occur with increases in workload and stress (see Table 2).

**Table 2:** Predicted and observed changes in experience from T1 to T2.

**Experience**	**Predicted changes**	**Observed findings**
Subjective wellbeing composite	Decrease	Decrease^†^
Stress	Increase	Increase
Health behaviors composite	Decrease	Decrease
Time on coursework	Increase	No change
Social activity composite	Decrease	No change

^†^A significant decrease in subjective wellbeing was observed in the planned t-tests but not in the LCSM.

##### 1.2.1.3 H3: Correspondence between personality and experience

Below we highlight previous research supporting a relationship between personality and the experiences measured in this study. For each trait-experience association specified below, we predict that (a) the trait at T1 will correlate with the experience at T1, (b) the trait at T2 will correlate with the experience at T2, and (c) changes in the trait from T1 to T2 will be associated with changes in the experience from T1 to T2. See Table 3 for a summary of these predictions.

**Table 3:** Predicted correlations between personality and experience and changes in personality and changes in experience.

**Experience**	**C**	**N**	**E**	**O**	**A**
**1. Subjective wellbeing**					
Composite		–	+		
Stressed		+	–		
Anxious		+	–		
Sad		+	–		
Happy		–	+		
Life satisfaction	+	–	+		
**2. Social support**					
Composite		–	+		+
Socially supported		–	+		+
Satisfied with relationships		–	+		+
Conflict in relationships		+	–		–
**3. Health behaviors**					
Composite	+	–			
Healthy eating	+				
Exercise	+	–	+		
Trouble sleeping	–	+			
Tiredness	–	+			
Sickness		+			
**4. Academic attendance and effort**					
Composite (standardized)	+				
Time in class	+				
Time on coursework	+			+	
Class absences	–	+	+		
**5. Extracurricular and work activities**					
Composite time (standardized)			+		
Time on extracurriculars			+		
Time working at job or internship	+		+		
Time spent volunteering			+		+
Trying new activities			+	+	
**6. Social activity**					
Composite (standardized)	–	–	+		+
Free time spent with friends	–	–	+		+
Time attending social events	–	–	+		+

Pluses denote a predicted positive correlation and minuses a predicted negative correlation. Because this table summarizes the predicted personality-experience correlations at T1, T2, and T1–T2, and there were some discrepancies in the observed pattern of correlations across time points, cells were shaded if the results supported the predictions for 2 of the 3 analyses.

###### 1.2.1.3.1 Conscientiousness

Based on previous research, we predict that conscientiousness will positively correlate with life satisfaction (Hayes and Joseph, 2003); healthy eating, exercise frequency, and overall health behaviors (Atherton et al., 2014; Kroencke et al., 2019; Wilson and Dishman, 2015); overall academic effort (Noftle and Robins, 2007; Trautwein et al., 2015), and time in class, on coursework (Bleidorn, 2012; Mehl et al., 2006), and working for a job (Wrzus et al., 2016). We predict that conscientiousness will negatively correlate with class absences, trouble sleeping, tiredness (Duggan et al., 2014), time with friends, time attending social events, and overall social activity (Mehl et al., 2006; Wrzus et al., 2016).

###### 1.2.1.3.2 Neuroticism

Based on previous research, we predict that neuroticism will negatively correlate with happiness, life satisfaction, and overall subjective wellbeing (Hayes and Joseph, 2003; Heller et al., 2007; Vaidya et al., 2002); support and satisfaction in relationships, time with friends and attending social events, and overall social support and activity (Lahey, 2009; Ozer and Benet-Martinez, 2006; Wrzus et al., 2016); and exercise frequency (Kroencke et al., 2019; Wilson and Dishman, 2015) and overall health behaviors (Atherton et al., 2014; Denney and Frisch, 1981; Williams and Wiebe, 2000). We also predict that neuroticism will positively correlate with stress, anxiety, sadness (Hayes and Joseph, 2003; Heller et al., 2007; McCrae and Costa Jr, 1991; Soto, 2019; Watson and Clark, 1984; Vaidya et al., 2002; Vollrath, 2000), conflict in relationships (Soto, 2019; Ozer and Benet-Martinez, 2006), trouble sleeping, tiredness (Duggan et al., 2014), sickness, and class absences (Løset and von Soest, 2023; Raynik et al., 2020).

###### 1.2.1.3.3 Extraversion

Based on prior research, we predict that extraversion will positively correlate with happiness, life satisfaction, and overall subjective wellbeing (Hayes and Joseph, 2003; Heller et al., 2007; Soto, 2019; Vaidya et al., 2002; Wilt et al., 2011); support and satisfaction in relationships, time with friends and attending social events, and overall social support and activity (Eaton and Funder, 2003; Emmons et al., 1986; Ozer and Benet-Martinez, 2006; Soto, 2019; Vollrath, 2000; Wrzus et al., 2016); exercise frequency (Kroencke et al., 2019; Wilson and Dishman, 2015); class absences (Løset and von Soest, 2023); and time on extracurriculars, working for a job, volunteering, and trying new activities (Gocłowska et al., 2019; Ozer and Benet-Martinez, 2006; Soto, 2019). We predict that extraversion will negatively correlate with stress, anxiety, sadness, and conflict in relationships (Hayes and Joseph, 2003; Heller et al., 2007; McCrae and Costa Jr, 1991; Ozer and Benet-Martinez, 2006; Soto, 2019; Vaidya et al., 2002; Vollrath, 2000).

###### 1.2.1.3.4 Openness

Because openness is associated with enjoying intellectual activities (Wrzus et al., 2016) and variety and novelty in interests and experiences (Gocłowska et al., 2019; John and Srivastava, 1999; Mehl et al., 2006), we predict that those who score higher in openness will spend more time on coursework and be more likely to try new activities.

###### 1.2.1.3.5 Agreeableness

Based on prior research, we predict that agreeableness will negatively correlate with conflict in relationships and positively correlate with support and satisfaction in relationships, time with friends and attending events, overall social support and activity, and time volunteering (Ozer and Benet-Martinez, 2006; Soto, 2019; Wrzus et al., 2016).

##### 1.2.1.4 H4: Personality consistency

Because personality shows high levels of consistency over time (e.g., Atherton et al., 2021; Klimstra et al., 2018; Robins et al., 2001; Roberts and DelVecchio, 2000; Vaidya et al., 2002), increasing in consistency with shorter time intervals between measurements (Caspi et al., 2005), we expect personality at T1 to correlate strongly with personality at T2.

##### 1.2.1.5 H5: Consistency in experience

We expect experiences at T1 and T2 to correlate as in prior research (e.g., Vollrath, 2000).

## 2 Method

### 2.1 Participants and power analysis

We recruited participants during the Fall 2023 semester from the psychology participant pools at our institutions. We expected that the majority of our participants would be first-year students enrolled in introductory psychology but recruited students from all courses in the participant pool. At T1, the sample included 362 participants (241 women, 103 men, 16 other, 2 unreported; *M*_age_ = 19.30, *SD* = 3.57; 21.2% Hispanic; 1.7% American Indian or Alaska Native, 13.6% Asian, 9.7% Black or African American, 55% White, 20% Other; 22.8% conservative, 20.6% moderate, 42.4% liberal, 14.2% don't know). One hundred and ninety-four students were from Arizona State University, 95 from Towson University, and 71 from Hobart and William Smith Colleges. 58% of the sample were first years, 71.4% were residential students, and 9.7% were transfer students. Of these participants, 282 returned at T2 (77.9% retention); 141 were from Arizona State University (72.7% retention), 78 from Towson University (82.1% retention), and 63 from Hobart and William Smith Colleges (88.7% retention). An a priori power analysis indicated that *N* = 327 is needed to detect small changes in personality (*d* = 0.20) with 95% power at α = 0.05. We set the power high to ensure sufficient power to interpret null effects and powered for small effects as small-to-medium effects are observed in studies examining personality change over time (e.g., Atherton et al., 2021; Bleidorn, 2012; Robins et al., 2001). Because we anticipated dropouts from T1 to T2, we planned to recruit approximately double the sample size (i.e., *N* = 650) at T1. Although we were only able to recruit 362 participants within the first 3 weeks of the semester, our retention rate at T2 was much higher than expected, nearing our target sample size. However, our a priori power analysis should have used a corrected alpha of 0.01 for our primary hypothesis tests because we planned to run five tests, one for each Big Five trait. Therefore, we performed a sensitivity power analysis for total sample that completed both Parts 1 and 2 (*N* = 280).^1^ This analysis indicated that the study was adequately powered to detect *d* = 0.25 with 95% power (and *d* = 0.21 at 80% power) at α = 0.01.

### 2.2 Materials and procedure

Participants signed up to participate in a two-part online study during weeks 2–3 (T1) and 8–9 (T2) of the semester. At each time point, participants completed the measures below.

#### 2.2.1 Personality traits

##### 2.2.1.1 Big Five Inventory (BFI)

Participants completed the 44-item BFI (John and Srivastava, 1999) by rating the extent to which each statement described them *in the past week* (1 = *disagree strongly*, 5 = *agree strongly*). We asked participants to rate their personality over the past week to assess their perceptions of their personality during that time, rather than capture their general view of themselves. Alphas reached the preregistered 0.70 benchmark for each trait except openness (see Table 4), which was lower than expected (α = 0.65 at T1 and 0.63 at T2) due to low item-total correlations for two negatively phrased items. To maintain the integrity of the validated scale, no items were removed. Measures were created by averaging the items for each trait separately at T1 and T2.

**Table 4:** Observed changes in personality from T1 to T2.

	**T1**			**T2**			***t***	***p***	***d***

**Trait**	α	***M***	***SD***	α	***M***	***SD***			
Conscientiousness	0.78	3.52	0.67	0.83	3.28	0.72	−7.06^*^	<0.001	−0.42
Neuroticism	0.83	3.14	0.79	0.84	3.24	0.80	2.07	0.04	0.12
Extraversion	0.82	3.09	0.78	0.84	3.07	0.77	−0.60	0.55	−0.04
Openness	0.65	3.26	0.51	0.63	3.18	0.49	−2.60^*^	<0.01	−0.16
Agreeableness	0.70	3.88	0.55	0.79	3.70	0.65	−5.71^*^	<0.001	−0.34

^*^Indicates a statistically significant effect at α = 0.01, the corrected significance level for these primary tests of our hypotheses.

#### 2.2.2 Experiences

For each domain of experiences, if the items were internally consistent (α ≥ 0.70), we planned to analyze them as both a composite and separately to test our predictions (see Table 3). Although the items for some domains did not meet this threshold, in some cases, this appeared to be because the items tapped diverse aspects of a broad construct, rather than because they failed to capture a meaningful, underlying concept. Therefore, we analyzed each set of items as a composite when there was evidence (via positive item-total correlations) that the items were capturing a broader construct. For composite variables containing items measured in hours per week and/or on different response scales, individual items were standardized before computing composite scores, as preregistered. However, individual items reported in hours per week were left unstandardized when testing for experience change from T1 to T2, allowing us to compare each participant's T1 and T2 experiences directly, rather than their experiences relative to the mean at each time point.^2^

##### 2.2.2.1 Subjective wellbeing

Following Kroencke et al. (2019), participants self-reported components of their subjective wellbeing (Diener et al., 1999) by rating the extent to which they felt happy, sad, stressed, anxious, and satisfied with life in the past week (1 = *not at all*, 5 = *very much*; α = 0.74 at T1 and 0.78 at T2).

##### 2.2.2.2 Social support

Participants also rated the extent to which they felt socially supported, satisfied with their relationships, and conflict in their relationships in the past week (1 = *not at all*, 5 = *very much*; α = 0.59 at T1 and 0.67 at T2).

##### 2.2.2.3 Health behaviors

To assess health behaviors, participants rated how often they did each of the following in the past week (1 = *not at all*, 5 = *everyday*): ate healthy, exercised, had trouble sleeping, felt tired, and felt sick (adapted from Atherton et al., 2014; α = 0.57 at T1 and 0.64 at T2).

##### 2.2.2.4 Academic attendance and effort

Participants reported the number of hours in the past week they spent (1) attending class and (2) doing coursework outside of class (with a specification of what constitutes coursework). In addition, participants indicated how many absences from class they had in the past week.^3^ Items were standardized to create a composite measure (α = 0.37 at T1 and 0.33 at T2).

##### 2.2.2.5 Extracurricular and work activities

Participants estimated the number of hours they spent (1) on extracurricular activities, (2) working for a paid job, (3) working for an unpaid job or internship, and (4) volunteering in the past week, and reported if they tried any new opportunities or activities. Because the items measuring time spent on extracurricular/work activities appeared to be quite distinct (α = 0.22 at T1 and 0.19 at T2), we elected not to analyze them as a composite.

##### 2.2.2.6 Social activity

Participants also estimated the number of hours in the past week they spent having free time with friends and attending social gatherings (e.g., events and parties). Items were standardized to create a composite measure (α = 0.63 at T1 and 0.44 at T2).

#### 2.2.3 Demographics

Lastly, participants completed a demographic questionnaire assessing their age, gender identity, race, ethnicity, political orientation, year starting college and years completed, major, the current course(s) and number of courses/credit hours they are taking, institution, whether they transferred, and whether they are a residential or commuter student.

## 3 Results

We report descriptive statistics for all variables (see Tables 4, 5)^4^ and tested for differences between those who only completed T1 and those who completed both T1 and T2. Participants who only completed T1 scored slightly lower in conscientiousness at T1 (*M* = 3.35, *SD* = 0.62) than those who completed both parts (*M* = 3.52, *SD* = 0.67), *t*_(358)_ = −2.00, *p* = 0.046, *d* = −0.25. Participants who only completed T1 also scored lower in happiness (*M* = 3.51, *SD* = 0.94 vs. *M* = 3.83, *SD* = 0.92), *t*_(358)_ = −2.67, *p* = 0.01, *d* = −0.34, social support (*M* = 3.47, *SD* = 0.87 vs. *M* = 3.73, *SD* = 0.83), *t*_(358)_ = −2.42, *p* = 0.02, *d* = −0.31, and positive health behaviors (*M* = 3.03, *SD* = 0.72 vs. *M* = 3.23, *SD* = 0.73), *t*_(357)_ = −2.10, *p* = 0.04, *d* = −0.27, and higher in relationship conflict (*M* = 2.39, *SD* = 1.25 vs. *M* = 2.08 vs. *SD* = 1.11), *t*_(358)_ = 2.16, *p* = 0.03, *d* = 0.27, and sickness (*M* = 2.25, *SD* = 1.18 vs. *M* = 1.90, *SD* = 1.1), *t*_(357)_ = 2.46, *p* = 0.01, *d* = 0.31, at T1 than those who completed both parts. No other significant differences in personality or experiences emerged between participants who only completed T1 and those who completed both parts.

**Table 5:** Latent change parameters.

**Model**	**Parameter**	**β**	***b***	**Std. Error**	***p*-value**
Extraversion	Mean	−0.03	−0.02	0.05	0.64
	Variance	1	0.51	0.09	<0.001
Agreeableness	Mean	−0.43	−0.16	0.03	<0.001
	Variance	1	0.14	0.04	<0.001
Conscientiousness	Mean	−0.48	−0.25	0.04	<0.001
	Variance	1	0.27	0.06	<0.001
Neuroticism	Mean	0.13	0.11	0.06	0.05
	Variance	1	0.64	0.1	<0.001
Openness	Mean	−0.29	−0.13	0.04	<0.001
	Variance	1	0.2	0.05	<0.001
Subjective wellbeing	Mean	−0.11	−0.04	0.02	0.14
	Variance	1	0.1	0.04	0.01
Social support	Mean	−0.09	−0.06	0.05	0.23
	Variance	1	0.39	0.12	<0.001
Health behaviors	Mean	−0.45	−0.27	0.06	<0.001
	Variance	1	0.36	0.1	<0.001
Extracurricular	Mean	0.18	0.18	0.21	0.37
	Variance	1	1.05	1.59	0.51
Social activity	Mean	−0.18	−1.2	0.55	0.03
	Variance	1	44.98	24.02	0.06

Analyses at T1 included all participants, though we tested if the results differed when excluding participants who only completed T1. The overall pattern of results for T1 analyses remained the same when excluding these participants. However, a few non-significant personality-experience associations for extracurricular activities at T1 became significant (e.g., conscientiousness and time on extracurriculars, neuroticism and trying new activities, openness and working for an unpaid job/internship), and a few were no longer statistically significant (e.g., agreeableness and working for an unpaid job/internship).

### 3.1 Personality change

Latent Change Score Models (LCSM) and paired samples *t*-tests were conducted to assess change in each personality trait from T1 to T2. The LCSM were suggested in the review process and thus not preregistered. LCSM, a structural equation modeling approach, uses T1 to predict T2 and defines a latent change variable to capture the remaining variability not accounted for by the regression (Kievit et al., 2018; McArdle and Hamagami, 2001). Using the LCSM allows for an estimate of overall change as well as an estimate of the variability in change among participants. Working within the latent variable space also allows for removal of inherent measurement variance at the manifest variable level. Effect sizes were calculated for the LCSM (β) and for *t*-tests (*d*) to assess the strength of change. Because these analyses are the primary tests of the competing theories, we set alpha to 0.01 to control for family-wise error in performing five tests (one for each Big Five trait). We adopted the conventional alpha of 0.05 for all other analyses, which are secondary.

The overall results across the traits did not clearly support any of the three predicted theories (Table 1). Participants showed significant, small to moderate decreases in conscientiousness (β = −0.48, *d* = −0.42), agreeableness (β = −0.43, *d* = −0.34), and openness (β = −0.29, *d* = −0.16; see Table 4).^5^ There was no significant change in extraversion (β = −0.03, *d* = −0.04), and the increase in neuroticism (β = 0.13, *d* = 0.12) was not significant at the 0.01 alpha level.^6^ All latent change variables showed significant amounts of variance suggesting heterogeneity in the amount of change between T1 and T2 (see Tables 4, 5; for model fit statistics see Supplementary Table S11). In other words, despite the overall patterns of personality change observed across the sample, there was considerable variation among participants in how much their personality changed.

### 3.2 Experience change

Latent Change Score Models (LCSM) and paired samples *t-*tests were performed to test for changes in experience from T1 to T2. The results partially supported our predictions (Table 2). The *t*-tests showed a significant decline in subjective wellbeing and increase in stress from T1 to T2 (see Table 6). Although we did not specify predictions for the individual subjective wellbeing items other than stress, from T1 to T2, happiness and life satisfaction significantly decreased and anxiety and sadness showed no significant change (see Table 6). Also supporting our predictions, positive health behaviors significantly declined from T1 to T2; all positive health behaviors declined except healthy eating (see Table 6).

**Table 6:** Observed changes in experience from T1 to T2.

**Experience**	**T1**		**T2**		***t***	***p***	***d***
	***M***	***SD***	***M***	***SD***			
**1. Subjective wellbeing**							
Composite	3.11	0.78	2.99	0.81	−2.57^*^	0.01	−0.15
Stressed	3.55	1.12	3.76	1.06	3.03^*^	0.003	0.18
Anxious	3.45	1.22	3.41	1.21	−0.60	0.55	−0.04
Sad	2.76	1.13	2.86	1.16	1.35	0.18	0.08
Happy	3.83	0.92	3.64	1.01	−3.08^*^	0.002	−0.18
Life satisfaction	3.47	1.16	3.32	1.15	−2.08^*^	0.04	−0.12
**2. Social support**							
Composite	3.73	0.83	3.56	0.93	−3.05^*^	0.003	−0.18
Socially supported	3.62	1.09	3.50	1.13	−1.79	0.07	−0.11
Satisfied with relationships	3.63	1.15	3.48	1.20	−2.02^*^	0.05	−0.12
Conflict in relationships	2.08	1.11	2.31	1.25	2.97^*^	0.003	0.18
**3. Health behaviors**							
Composite	3.23	0.73	3.02	0.77	−5.54^*^	<0.001	−0.33
Healthy eating	3.07	1.06	3.01	1.07	−1.13	0.26	−0.07
Exercise	2.88	1.42	2.62	1.34	−3.65^*^	<0.001	−0.22
Trouble sleeping	2.46	1.24	2.73	1.27	3.65^*^	<0.001	0.22
Tiredness	3.46	1.11	3.69	1.07	3.38^*^	<0.001	0.20
Sickness	1.90	1.11	2.09	1.22	2.36^*^	0.02	0.14
**4. Academic attendance and effort**							
Composite (standardized)	−0.04	0.59	−0.003	0.59	0.24	0.81	0.02
Time in class	11.96	5.51	11.16	5.86	−1.84	0.07	−0.11
Time on coursework	12.48	11.00	12.23	9.44	−0.42	0.68	−0.03
Class absences	0.34	0.91	0.74	1.38	4.48^*^	<0.001	0.27
**5. Extracurricular and work activities**							
Composite time (standardized)	–	–	–	–	–	–	–
Time on extracurriculars	4.81	5.68	5.59	6.72	2.30^*^	0.01	0.14
Time working at paid job/internship	5.60	10.06	5.39	9.42	−0.55	0.58	−0.03
Time working at unpaid job/internship	0.17	1.34	0.31	1.74	1.19	0.24	0.07
Time spent volunteering	0.31	1.11	0.50	1.69	1.92^*^	0.06	0.12
Trying new activities	0.56	0.49	0.40	0.49	−4.13^*^	<0.001	−0.25
**6. Social activity**							
Composite (standardized)	−0.01	0.83	−0.003	0.80	0.20	0.84	0.01
Free time spent with friends	9.87	9.77	8.65	8.71	−2.06^*^	0.04	−0.12
Time attending social events	3.47	3.71	3.14	4.12	−1.12	0.26	−0.07

^*^Indicates a statistically significant effect at α = 0.05.

Counter to our predictions, there was no significant increase in time on coursework. Exploratory analyses showed no changes in overall academic effort or time in class. However, class absences significantly increased from T1 to T2 (see Table 6). Also counter to our predictions, the composite measure of social activity showed no significant decline, though participants reported significantly less time spent with friends from T1 to T2 (see Table 6).

We did not specify predictions for changes in social support or time on extracurriculars. Exploratory analyses found a significant decrease in composite social support and relationship satisfaction, and significant increase in relationship conflict. Time on extracurriculars increased from T1 to T2, trying new activities decreased, and there was no significant change in time volunteering or working for a paid or unpaid job or internship (see Table 6).

The LCSM indicated a significant decline in positive health behaviors (β = −0.45, *p* < 0.001), consistent with the *t*-tests, and a significant decrease in social activity (β = −0.18, *p* = 0.03). All other latent models did not show significant change in the experience behaviors. However, the variability in change was significant for the majority of these constructs between T1 and T2, suggesting individual differences in change over time (see Table 5).

### 3.3 Correspondence between personality and experience

Correlations were conducted between the personality traits and experiences at each time point (Tables 7, 8), and personality change scores and experience change scores (Table 9). For each predicted trait-experience association, we expected the trait at T1 would correlate with the experience at T1, the trait at T2 would correlate with the experience at T2, and changes in the trait from T1 to T2 would be associated with changes in the experience from T1 to T2 (Table 3).^7^ Factor scores were extracted from each latent change variable for personality and experiences, and correlations were calculated among the factor scores (Table 10).

**Table 7:** Observed correlations between personality and experience at T1.

**Experience**	**C**	**N**	**E**	**O**	**A**
**1. Subjective wellbeing**					
Composite	0.30^*^	−0.73^*^	0.37^*^	0.06	0.32^*^
Stressed	−0.21^*^	0.54^*^	−0.12^*^	0.05	−0.13^*^
Anxious	−0.15^*^	0.64^*^	−0.19^*^	0.09	−0.11^*^
Sad	−0.20^*^	0.55^*^	−0.17^*^	0.03	−0.23^*^
Happy	0.21^*^	−0.42^*^	0.45^*^	0.20^*^	0.39^*^
Life satisfaction	0.30^*^	−0.39^*^	0.39^*^	0.19^*^	0.30^*^
**2. Social support**					
Composite	0.30^*^	−0.43^*^	0.37^*^	0.13^*^	0.34^*^
Socially supported	0.24^*^	−0.28^*^	0.43^*^	0.19^*^	0.25^*^
Satisfied with relationships	0.22^*^	−0.33^*^	0.38^*^	0.11^*^	0.23^*^
Conflict in relationships	−0.22^*^	0.34^*^	−0.02	0.02	−0.28^*^
**3. Health behaviors**					
Composite	0.44^*^	−0.38^*^	0.26^*^	0.03	0.29^*^
Healthy eating	0.35^*^	−0.23^*^	0.20^*^	0.04	0.14^*^
Exercise	0.24^*^	−0.12^*^	0.15^*^	−0.04	0.06
Trouble sleeping	−0.26^*^	0.23^*^	−0.12^*^	−0.04	−0.26^*^
Tiredness	−0.26^*^	0.40^*^	−0.20^*^	−0.02	−0.19^*^
Sickness	−0.27^*^	0.23^*^	−0.13^*^	−0.05	−0.23^*^
**4. Academic attendance and effort**					
Composite (standardized)	−0.001	0.14^*^	−0.03	0.04	−0.01
Time in class	0.06	0.05	0.05	−0.04	0.03
Time on coursework	0.15^*^	0.10	−0.07	0.11^*^	0.05
Class absences	−0.22^*^	0.10	−0.03	0.001	−0.11^*^
**5. Extracurricular and work activities**					
Composite time (standardized)	–	–	–	–	–
Time on extracurriculars	0.10	−0.03	0.13^*^	0.10	0.02
Time working paid job/internship	0.11^*^	−0.04	0.03	0.01	−0.02
Time working unpaid job/internship	−0.01	−0.001	0.04	0.06	−0.15^*^
Time spent volunteering	−0.01	−0.01	0.01	0.09	0.02
Trying new activities	0.13^*^	−0.08	0.16^*^	0.11^*^	0.17^*^
**6. Social activity**					
Composite (standardized)	0.01	−0.12^*^	0.32^*^	−0.01	0.10
Free time spent with friends	0.01	−0.08	0.22^*^	−0.04	0.07
Time attending social events	−0.004	−0.13^*^	0.33^*^	0.02	0.10

p <0.05.

**Table 8:** Observed correlations between personality and experience at T2.

**Experience**	**C**	**N**	**E**	**O**	**A**
**1. Subjective wellbeing**					
Composite	0.38^*^	−0.81^*^	0.51^*^	0.20^*^	0.43^*^
Stressed	−0.18^*^	0.59^*^	−0.21^*^	−0.07	−0.22^*^
Anxious	−0.23^*^	0.60^*^	−0.17^*^	−0.11	−0.20^*^
Sad	−0.34^*^	0.67^*^	−0.38^*^	−0.11	−0.32^*^
Happy	0.26^*^	−0.57^*^	0.58^*^	0.24^*^	0.46^*^
Life satisfaction	0.35^*^	−0.51^*^	0.53^*^	0.20^*^	0.37^*^
**2. Social support**					
Composite	0.35^*^	−0.45^*^	0.49^*^	0.20^*^	0.43^*^
Socially supported	0.28^*^	−0.35^*^	0.51^*^	0.20^*^	0.32^*^
Satisfied with relationships	0.34^*^	−0.36^*^	0.46^*^	0.15^*^	0.33^*^
Conflict in relationships	−0.20^*^	0.35^*^	−0.18^*^	−0.11	−0.34^*^
**3. Health behaviors**					
Composite	0.47^*^	−0.57^*^	0.39^*^	0.11	0.36^*^
Healthy eating	0.45^*^	−0.41^*^	0.33^*^	0.08	0.24^*^
Exercise	0.32^*^	−0.21^*^	0.27^*^	−0.02	0.12
Trouble sleeping	−0.26^*^	0.41^*^	−0.21^*^	−0.08	−0.33^*^
Tiredness	−0.24^*^	0.51^*^	−0.33^*^	−0.16^*^	−0.24^*^
Sickness	−0.24^*^	0.32^*^	−0.15^*^	−0.06	−0.24^*^
**4. Academic attendance and effort**					
Composite (standardized)	0.02	0.09	−0.03	−0.02	0.01
Time in class	0.09	−0.06	0.03	0.06	0.04
Time on coursework	0.18^*^	0.08	0.01	−0.002	0.002
Class absences	−0.23^*^	0.13^*^	−0.08	−0.09	−0.04
**5. Extracurricular and work activities**					
Composite time (standardized)	–	–	–	–	–
Time on extracurriculars	0.17^*^	−0.11	0.21^*^	0.14^*^	0.08
Time working paid job/internship	0.10	0.002	0.07	−0.06	−0.05
Time working unpaid job/internship	−0.03	−0.06	0.04	0.06	−0.06
Time spent volunteering	<0.001	−0.02	0.06	0.07	0.02
Trying new activities	0.12^*^	−0.16^*^	0.14^*^	0.21^*^	0.08
**6. Social activity**					
Composite (standardized)	0.02	−0.16^*^	0.27^*^	0.02	0.12
Free time spent with friends	−0.01	−0.15^*^	0.23^*^	0.04	0.11
Time attending social events	0.04	−0.10	0.21^*^	0.001	0.07

^*^p <0.05.

**Table 9:** Observed correlations between changes in personality and changes in experience.

**Experience**	**C**	**N**	**E**	**O**	**A**
**1. Subjective wellbeing**					
Composite	0.32^*^	−0.59^*^	0.44^*^	0.13^*^	0.27^*^
Stressed	−0.17^*^	0.37^*^	−0.15^*^	0.01	−0.13^*^
Anxious	−0.16^*^	0.38^*^	−0.24^*^	−0.01	−0.09
Sad	−0.27^*^	0.50^*^	−0.32^*^	−0.10	−0.20^*^
Happy	0.25^*^	−0.44^*^	0.46^*^	0.24^*^	0.36^*^
Life satisfaction	0.22^*^	−0.31^*^	0.32^*^	0.12^*^	0.16^*^
**2. Social support**					
Composite	0.19^*^	−0.30^*^	0.32^*^	0.19^*^	0.29^*^
Socially supported	0.11	0.05	0.25^*^	0.14^*^	0.15^*^
Satisfied with relationships	0.21^*^	0.10	0.27^*^	0.17^*^	0.14^*^
Conflict in relationships	−0.09	0.10	0.03	0.02	−0.12
**3. Health behaviors**					
Composite	0.34^*^	−0.25^*^	0.24^*^	0.10	0.27^*^
Healthy eating	0.20^*^	−0.14^*^	0.12^*^	0.05	0.21^*^
Exercise	0.14^*^	0.02	0.02	−0.12^*^	0.15^*^
Trouble sleeping	−0.26^*^	0.15^*^	−0.20^*^	−0.11	−0.11
Tiredness	−0.12^*^	0.27^*^	−0.20^*^	−0.12^*^	−0.12^*^
Sickness	−0.18^*^	0.11	−0.09	−0.09	−0.15^*^
**4. Academic attendance and effort**					
Composite (standardized)	0.01	0.02	−0.08	0.02	−0.03
Time in class	0.06	0.10	0.04	0.07	−0.02
Time on coursework	0.10	0.03	−0.10	−0.03	−0.04
Class absences	−0.15^*^	−0.09	−0.07	−0.003	0.001
**5. Extracurricular and work activities**					
Composite time (standardized)	–	–	–	–	–
Time on extracurriculars	0.11	−0.03	0.05	0.11	0.07
Time working paid job/internship	0.04	0.10	0.01	0.03	0.01
Time working unpaid job/internship	0.13^*^	−0.10	0.04	0.06	0.03
Time spent volunteering	−0.04	−0.01	−0.05	0.04	−0.01
Trying new activities	0.13^*^	−0.17^*^	0.15^*^	0.09	0.18^*^
**6. Social activity**					
Composite (standardized)	−0.05	−0.11	0.17^*^	0.02	0.04
Free time spent with friends	−0.01	−0.13^*^	0.17^*^	0.09	0.01
Time attending social events	−0.07	−0.04	0.10	−0.06	0.05

^*^p <0.05.

**Table 10:** Correlations among latent change variable factor scores.

**Latent Change Variable**		**1**	**2**	**3**	**4**	**5**	**6**	**7**	**8**	**9**
1.	Conscientiousness Δ									
2.	Neuroticism Δ	−0.26^*^								
3.	Extraversion Δ	0.22^*^	−0.33^*^							
4.	Openness Δ	0.29^*^	−0.17^*^	0.34^*^						
5.	Agreeableness Δ	0.20^*^	−0.29^*^	0.25^*^	0.18^*^					
6.	Subjective wellbeing Δ	0.29^*^	−0.57^*^	0.32^*^	0.08	0.20^*^				
7.	Social support Δ	0.20^*^	−0.26^*^	0.30^*^	0.21^*^	0.23^*^	0.27^*^			
8.	Health behaviors Δ	0.30^*^	−0.29^*^	0.24^*^	0.14^*^	0.17^*^	0.29^*^	0.11^*^		
9.	Extracurricular Δ	0.02	−0.01	0.00	0.09	0.00	−0.07	−0.01	−0.02	
10.	Social activity Δ	−0.03	−0.11^*^	0.17^*^	0.01	−0.02	0.06	0.10	0.05	0.01

^*^p <0.05.

#### 3.3.1 Subjective wellbeing

Results supported our predictions (see Tables 7–9): conscientiousness positively correlated with life satisfaction; neuroticism negatively correlated with happiness, life satisfaction, and subjective wellbeing and positively correlated with stress, anxiety, and sadness; and extraversion negatively correlated with stress, anxiety, and sadness and positively correlated with happiness, life satisfaction, and subjective wellbeing. At each time point, neuroticism showed the strongest correlations with subjective wellbeing, *r*s ≤ −0.59, and extraversion showed moderate positive correlations with subjective wellbeing. Although unpredicted, conscientiousness and agreeableness were also moderately positively associated with subjective wellbeing at each time point, and openness showed small positive correlations with subjective wellbeing at T2 and between openness change and subjective wellbeing change from T1 to T2 (see Tables 7–9). The pattern of results is supported by the correlations among the factor scores (see Table 10).

#### 3.3.2 Social support

Overall, results supported our predictions for social support (see Tables 7–9): neuroticism negatively correlated with composite social support and support and satisfaction in relationships, and positively correlated with relationship conflict. Extraversion positively correlated with composite social support and support and satisfaction in relationships, and agreeableness positively correlated with composite social support, support and satisfaction in relationships, and relationship conflict. However, extraversion was not significantly associated with relationship conflict at T1, and for the changes in personality associated with changes in social support, the results supported the predictions for the overall composite social support measure but varied for the individual social support items. Although we did not specify predictions for conscientiousness or openness, these variables positively correlated with social support at each time point as well (see Tables 7–9). The relationships among the factor scores of the latent change variables highlight similar findings (see Table 10).

#### 3.3.3 Health behaviors

In general, the results supported our predictions for health behaviors: conscientiousness positively correlated with positive health behaviors, healthy eating, and exercise and negatively correlated with trouble sleeping and tiredness. Neuroticism negatively correlated with positive health behaviors and exercise and positively correlated with trouble sleeping, tiredness, and sickness. Extraversion positively correlated with exercise frequency. However, changes in neuroticism and changes in extraversion were not significantly correlated with changes in exercise, and changes in neuroticism were not significantly associated with changes in sickness (see Tables 7–9). Although unpredicted, extraversion was positively associated with overall positive health behaviors and healthy eating and negatively associated with trouble sleeping and tiredness at each time point. Conscientiousness was also negatively correlated with sickness at each time point. The relationships among the latent change variables highlighted similar patterns (see Table 10).

#### 3.3.4 Academic effort

As predicted, conscientiousness was positively correlated with time on coursework at T1 and T2 and negatively correlated with class absences at each time point (see Tables 7–9). However, counter to our predictions, conscientiousness was not significantly correlated with overall academic effort or time in class, and changes in conscientiousness were unrelated to changes in time on coursework (see Tables 7–9). Also counter to our hypotheses, neuroticism was not significantly correlated with class absences at T1 and changes in neuroticism were not significantly related to changes in absences; however, neuroticism did show a small, significant positive correlation with absences at T2. Extraversion was not significantly related to absences at any time point. In partial support of our hypotheses, openness was positively correlated with time on coursework at T1; however, openness was not significantly related to time on coursework at T2 and change in openness was not significantly correlated with change in time on coursework (see Tables 7–9). A latent change score model for academic effort did not converge and cannot be included in the correlation matrix.

#### 3.3.5 Extracurriculars and work activities

Results partially supported our predictions for extracurricular/work activities: There was a small, significant correlation between conscientiousness and time working for a job/internship at T1 but not T2, and changes in conscientiousness were not significantly correlated with changes in time working (see Tables 7–9). Extraversion showed small, positive correlations with time on extracurriculars and trying new activities at all time points, except that changes in extraversion were not significantly correlated with changes in time spent on extracurriculars. Openness was positively correlated with trying new activities at T1 and T2, but changes in openness were not significantly correlated with changes in trying new activities. Counter to our predictions, agreeableness was not significantly related to time volunteering at any time point (see Tables 7–9). Although unpredicted, agreeableness was positively correlated with trying new activities at T2, and changes in agreeableness were positively correlated with changes in trying new activities. The factor scores for the extracurricular/work activities change variable was not significantly related to any other changes that were measured (*r*s <|0.07|; see Table 10).

#### 3.3.6 Social activity

Counter to our predictions, conscientiousness and agreeableness were not significantly associated with social activity, time with friends, or time attending social events at any time point (see Tables 7–9). In partial support of our hypotheses, neuroticism was significantly negatively correlated with social activity and time attending social events at T1 (but not time with friends), social activity and time with friends at T2 (but not time attending social events), and changes in neuroticism were significantly negatively correlated with changes in time with friends (but not social activity or time attending social events). Supporting our predictions, extraversion was positively associated with social activity, time with friends, and time attending social events at all time points, except that extraversion change was not significantly correlated with change in time attending social events. The relationships among the latent change factor scores showed similar patterns with changes in social activity being negatively related to change in neuroticism and positively with change in extraversion.

### 3.4 Personality and experience consistency

Correlational analyses were performed to test the consistency of each personality trait and experience from T1 to T2. As predicted, personality at T1 strongly correlated with personality at T2 for each trait, *r*s ≥ 0.54, *p*'s <0.001 (see Table 11). At T1 and T2, subjective wellbeing correlated strongly, *r* = 0.53, *p* < 0.001, social support correlated moderately to strongly, *r* = 0.45, *p* < 0.001, positive health behaviors correlated strongly, *r* = 0.67, *p* < 0.001, academic effort correlated slightly to moderately, *r* = 0.27, *p* < 0.001, time on extracurriculars correlated strongly, *r* = 0.60, *p* < 0.001, time working for a paid job or internship correlated strongly, *r* = 0.79, *p* < 0.001, time working for an unpaid job/internship correlated weakly, *r* = 0.20, *p* < 0.001, time volunteering correlated moderately, *r* = 0.40, *p* < 0.001, trying new activities correlated weakly, *r* = 0.17, *p* = 0.004, and social activity correlated moderately, *r* = 0.38, *p* < 0.001. See the Supplementary material for correlations among the individual items of each composite measure.

**Table 11:** Correlations between personality traits at T1 and T2.

**Trait**	**C, T1**	**N, T1**	**E, T1**	**O, T1**	**A, T1**	**C, T2**	**N, T2**	**E, T2**	**O, T2**
Conscientiousness, T1	–								
Neuroticism, T1	−0.32^*^	–							
Extraversion, T1	0.17^*^	−0.29^*^	–						
Openness, T1	0.19^*^	−0.06	0.24^*^	–					
Agreeableness, T1	0.34^*^	−0.40^*^	0.22^*^	0.24^*^	–				
Conscientiousness, T2	**0.66** ^*^	−0.27^*^	0.21^*^	0.08	0.35^*^	–			
Neuroticism, T2	−0.27^*^	**0.54** ^*^	−0.25^*^	−0.002	−0.34^*^	−0.42^*^	–		
Extraversion, T2	0.20^*^	−0.23^*^	**0.63** ^*^	0.17^*^	0.23^*^	0.34^*^	−0.49^*^	–	
Openness, T2	0.13^*^	−0.10	0.14^*^	**0.54** ^*^	0.21^*^	0.18^*^	−0.22^*^	0.35^*^	–
Agreeableness, T2	0.31^*^	−0.31^*^	0.22^*^	0.14^*^	**0.59** ^*^	0.37^*^	−0.49^*^	0.38^*^	0.25^*^

^*^p <0.05. Bolded correlations highlight consistency between T1 and T2 personality measures.

### 3.5 Robustness analyses

For all confirmatory analyses, robustness checks were performed testing for possible moderating effects of age and gender,^8^ and exploratory analyses tested for moderating effects of year in college, institution, and residential vs. commuter student status. Differences may be stronger among first years, as it is a transitional period with the most strain (Bewick et al., 2010).

Neither gender nor age significantly moderated any of the personality change effects. Year in college (first year vs. non-first year), institution, and residential vs. commuter student status also did not moderate the personality change effects.

## 4 Discussion

### 4.1 Personality change

In this study, we tested competing theories of personality change across the academic semester and examined the experiences associated with personality change. The situational perspective predicted that conscientiousness and extraversion would decline and neuroticism would increase in response to the increasing demands across the semester; the maturity principle predicted that conscientiousness and agreeableness would increase and neuroticism would decrease as students adapt to new roles and responsibilities; and the personality stability perspective predicted no change, as personality shows high stability, especially within short time intervals (Caspi et al., 2005).

The results of this study did not clearly support any of these theories. We observed a decrease in conscientiousness, consistent with the situational perspective, but also a decrease in openness and agreeableness, which were unpredicted from any of the competing hypotheses. We did not observe significant changes in neuroticism and extraversion, but further research is needed to conclusively determine whether neuroticism and extraversion change across the semester. It is possible that neuroticism and extraversion would more closely follow the predicted changes from the situational perspective in a larger sample, as the findings were directionally consistent with the predictions from this perspective. In addition, although we had a relatively high retention rate (77.9%), we could only test for personality change among those who completed both parts of the study, and thus our results might not generalize to the wider student population.

The new expectations, roles, and environment associated with college may challenge college students, resulting in short-term declines in conscientiousness, agreeableness, and openness during this transition. In this sense, the results may reflect a different version of the situational perspective. From the situational perspective, we had expected conscientiousness and extraversion to decline and neuroticism to increase across the semester due to increasing demands, as prior between-subject studies had found lower conscientiousness, less positive mood, and higher stress among study participants later vs. earlier in the semester (Aviv et al., 2002; Ebersole et al., 2016; Witt et al., 2011). We theorized that individuals may decrease rather than increase in conscientiousness and increase rather than decrease in neuroticism in the shorter-term across the semester before adapting and changing from these experiences. The results suggest this may be the case, but for somewhat different traits than predicted (i.e., conscientiousness, openness, and agreeableness rather than conscientiousness, neuroticism, and extraversion). We had not expected to see drops in openness and agreeableness, but students may be more open to experiences and agreeable at the beginning of the semester when facing less stress, demands, and challenges, and focusing more on making friends and exploring a new environment. Future research is needed to replicate and better understand whether these short-term patterns of personality change observed across the semester differ from long-term patterns of personality change found over the college years (e.g., Atherton et al., 2021; Klimstra et al., 2018).

For all traits, we observed significant variability in how much participants' personality changed across the semester. In other words, despite the change (or lack of change) observed across participants for each trait, there was significant variation among participants in the degree of personality change. These findings may suggest that some individuals experience more personality change in response to situational pressures than others. Future research should seek to examine predictors and outcomes associated with the degree of personality stability vs. change individuals exhibit.

### 4.2 Experience change

This study also tested for changes in affective, health, academic, extracurricular, and social experiences over the semester. Supporting prior research (e.g., Fuller et al., 2003; Kroencke et al., 2019; Pitt et al., 2018) and our predictions, the planned paired samples *t*-tests showed a decrease in overall subjective wellbeing, increase in stress, and decrease in health behaviors. Participants did not show significant changes in anxiety or sadness but did show declines in happiness and life satisfaction. In addition, participants showed declines in all health behaviors except healthy eating.

Counter to our predictions, we observed no change in time on coursework across the semester. However, class absences significantly increased and time spent with friends significantly decreased over the semester. One potential explanation for why time on coursework might not have increased over the semester is that since the COVID-19 pandemic, professors may have shifted toward a series of small, low-stakes assignments rather than fewer, higher stakes assessments, and thus the workload may be more evenly distributed throughout the semester. Additional research is needed to further investigate this unexpected finding.

Exploratory analyses performed on the other experience variables showed a significant decline in social support from T1 to T2, increase in time on extracurriculars, decrease in trying new activities, and no significant change in time working for a job or internship. Overall, these declines in variables related to psychological wellbeing (e.g., affect, health, social support, time with friends) may suggest that the academic semester is a challenging and demanding time for students, placing strain on many areas of their lives. However, the effect sizes for these experience changes were small, and the LCSM did not show significant changes in experiences except for health behaviors and social activity,^9^ which questions the robustness of these findings. The low reliability for several of the experience change variables might have limited the ability to detect changes and should be addressed in future research. As with the personality change, there was significant variability in experience change across participants, which warrants further investigation in future research.

### 4.3 Correspondence between personality and experience

In addition to changes in personality and experience across the semester, we examined how changes in personality corresponded with changes in experience, and how personality related to experience at each time point. All traits except openness were associated with subjective wellbeing at T1, all were related to subjective wellbeing at T2, and increases in conscientiousness, extraversion, openness, and agreeableness and decreases in neuroticism were associated with increases in subjective wellbeing from T1 to T2. As in prior research, neuroticism and to a lesser extent extraversion showed the strongest association with subjective wellbeing at each time point (e.g., Hayes and Joseph, 2003; Soto, 2019). However, because previous research has not found consistent links between agreeableness and openness and subjective wellbeing (e.g., Hayes and Joseph, 2003; Soto, 2019), further research is needed to determine whether the observed relationships between subjective wellbeing and these traits are specific to the sample and time period studied (i.e., college students across the semester).

All Big Five traits were also associated with social support, all traits but openness were associated with positive health behaviors, and changes in the traits (except openness) were associated with changes in social support and positive health behaviors. As predicted, conscientiousness positively correlated with time on coursework at T1 and T2 and negatively with class absences at each time point. Overall academic effort was not significantly related to any of the traits, except for neuroticism at T1, and changes in academic effort were not significantly related to changes in any of the traits. These findings may be due to the low reliability of the composite academic effort measure. In addition, changes in academic effort within a semester may be small and not closely related to changes in personality in the short-term, but consistent changes in academic effort over the college years may be associated with long-term personality change, such as an increase in conscientiousness.

Time on extracurriculars correlated with extraversion, consistent with our predictions; however, the strength of this correlation was small. Conscientiousness was only significantly related to time working for a paid job or internship at T1, and this relationship was also weak. However, increases in time spent working for an unpaid job or internship were associated with increases in conscientiousness. Time spent working for a paid job varied widely among participants (from 0 to 60+ hours per week). This variability may be more strongly related to the extent to which college students need to work during the semester due to their financial situation, rather than to differences in personality.

Trying new activities showed small correlations with many of the personality traits at different time points, though surprisingly, openness to experience only showed a modest correlation with trying new activities at T2. Supporting previous research (e.g., Ozer and Benet-Martinez, 2006; Soto, 2019; Wrzus et al., 2016) and our predictions, social activity was positively associated with extraversion at each time point, and negatively associated with neuroticism at T1 and T2. However, social activity was not significantly correlated with other personality traits such as agreeableness and conscientiousness, in contrast to previous research (e.g., Wrzus et al., 2016).

Together, these findings support prior research indicating that personality relates to numerous important behaviors and life outcomes (e.g., Ozer and Benet-Martinez, 2006; Soto, 2019), though not all predicted personality-experience associations were supported. The personality-experience correlations for subjective wellbeing, social support, and health behaviors tended to be somewhat stronger at T2 than T1, possibly indicating that these associations increase in strength under situational pressure. Changes in personality co-occurred with changes in experiences, particularly subjective wellbeing, social support, and health behaviors, suggesting that personality and these experiences are interlinked.

### 4.4 Personality and experience consistency

Supporting research on the rank-order consistency of personality (Roberts and DelVecchio, 2000), we found high levels of personality consistency from T1 to T2. We also found moderate to strong consistency in most experiences, including subjective wellbeing, health behaviors, social support, and social activity. Time on coursework, time on extracurriculars, time working for a paid job or internship were strongly correlated at T1 and T2, though the other academic effort and extracurricular/work variables showed more modest correlations. These findings, combined with our personality and experience change results, provide evidence of both strong personality and behavior consistency and personality and behavior change in response to the situation.

### 4.5 Limitations, implications, and directions for future research

There were some limitations to this research that provide important avenues for future research. One limitation was the low reliability of some of the composite measures of experience. In some cases, these low reliabilities may reflect weak interrelationships among the items. For example, most of the individual time on extracurricular and work activity items were not significantly correlated. In other cases, lower than ideal reliability may reflect the broad nature of the overarching construct (e.g., health behaviors), rather than a lack of relation or poor measurement. Regardless of the reason, because observed correlations are constrained by the reliabilities of the variables (Nunnally, 1970), the true relationships between personality and experiences may be stronger than those observed in the present research. Indeed, personality tended to correlate more strongly with the experience measures with higher reliability. In addition, two negatively phrased items on our openness measure had negative item-total correlations, which might have reduced the strength of results for this variable. Even so, we did observe consistent findings for some of the individual items (e.g., class absences, trying new activities), even when the composite measure had low reliability. We also observed strong correlations for some variables (e.g., health behaviors, social support, and social activity), even with lower than ideal composite reliability.

Prior research and theory support many of the personality-experience associations observed in this research. However, we caution against drawing strong conclusions from these associations, as some statistically significant correlations may be false positives due to the number of correlations performed. Future research replicating these findings is needed before drawing strong conclusions from these associations.

Furthermore, because the primary personality change effects were unpredicted, it is important to replicate this study to further understand the short and long-term patterns of personality change that occur among college students across the semester vs. over the college years. Although the results did not clearly support any of our a priori hypotheses, the data partially support a fourth alternative hypothesis we had not considered: the disruption hypothesis. The disruption hypothesis suggests that individuals experience temporary dips in traits associated with psychological maturity as they transition from childhood to adolescence (Soto and Tackett, 2015). Studies have found that conscientiousness, agreeableness, and openness levels decrease from late childhood to early adolescence before increasing from late adolescence to early adulthood (Soto et al., 2011; see Table 1). These are the trends we observed in the present study across the academic semester. We had not considered the relevance of this hypothesis to our current sample, given its focus in the literature on youth personality development from late childhood to early adolescence. However, it is possible that college students show a pattern of disruption in the transition to adulthood and independence before fully displaying maturity. To fully test whether college students exhibit patterns of disruption followed by maturation, researchers would need to measure personality at a third later time point to assess whether these short-term changes rebounded and reversed in direction in the longer-term.

To better understand these different patterns of personality change, future research could track participants at the beginning and two-thirds of the way through the Fall semester, as in the present research, and at the beginning of the Spring semester, as in Bleidorn (2012), to examine whether short-term changes in traits within the semester reflecting disruption do indeed differ from changes reflecting personality growth from the beginning of one semester to the next. Studies could also compare change from the beginning to middle-end of the Fall semester with change from the beginning to end of an academic year. In addition, replicating the findings across the Fall semester in the Spring, or comparing changes across a similar time frame in a non-college sample, would help demonstrate whether the short-term changes are due to changes in experiences during the semester rather than due to seasonal changes. Future studies may consider using the more recently updated version of the Big Five Inventory (Soto and John, 2017), rather than the more dated one used in the present research (John and Srivastava, 1999), and more reliable measures of experience. The experience change variables measured in hours per week appeared to be the least reliable; using a different response scale may help reduce measurement error.

The personality change effects observed in this study were unaffected by gender, age, participants' year in college, which institution they attended, and whether they were a residential or commuter student. Although no significant interactions emerged with gender or age, because the study was not powered to test for interactive effects and prior studies have shown age and gender differences in personality and personality change (e.g., Roberts and Mroczek, 2008; Roberts et al., 2006; Soto et al., 2011; Weisberg et al., 2011), future studies may wish to further explore these variables in a larger sample. In addition, while the universities sampled in this study were diverse in several ways (e.g., size, location, type of institution) and the personality changes did not differ by university, future studies could replicate these findings at other universities, including in other countries, that further vary in student body demographics and academic culture.

We asked participants to rate their personality in the past week to assess their personality during that time period, rather than how they generally see themselves. This approach is typically used in research on personality states, in which participants rate themselves over a specific time interval (Fleeson, 2001, 2007; Heller et al., 2007). However, it is possible that this retrospective approach to measuring personality is unreliable and/or exaggerated differences based on misperceptions and faulty recall. Future research is needed to understand whether self-reports of personality vary when asked to rate personality over short, specific time periods compared to in general or without specifying.

Although little prior research has examined personality change in time periods between days and years, the relatively modest effect sizes observed in this research are consistent with those found in studies done in similar time frames (e.g., Bleidorn, 2012). These findings may have the implication that the impact of administering studies at different times of the semester would be relatively small, based on personality. However, to participate in this study, participants had to sign up within the first few weeks of the semester. Thus, the characteristics of those who get started early on their research participation requirement may differ from those who did not sign up in time to participate. In addition, we found small differences between those who dropped out after T1 and those who completed both parts, with dropouts scoring lower in conscientiousness, happiness, social support, and positive health behaviors, and higher in relationship conflict and sickness at T1. These findings suggest that dropouts have somewhat different (and more negative) experiences than participants who returned at T2. Because the personality changes observed in this research may be specific to students who participate in studies early in the semester, future researchers may consider adopting different recruitment strategies (e.g., administering surveys to entire classes at the beginning of the semester) to avoid possible selection bias that may limit the generalizability of the findings.

### 4.6 Conclusion

Few studies have examined personality change over the span of the college semester. College is an important transitional period for many young adults, and students face new demands that can vary in intensity across the semester. Previous research assessed personality at the beginning of the first and second semester of the first year of college and observed an increase in conscientiousness, openness, and decrease in neuroticism (Bleidorn, 2012). We measured personality at the beginning and two-thirds of the way through the Fall semester and observed a decrease in conscientiousness, openness, and agreeableness. These diverging findings may be reflective of the disruption hypothesis, in which college students show short-term declines in traits associated with the maturity principle in response to new challenges and expectations before adapting and growing from those experiences. However, further research is needed to directly test this possibility. Future research should seek to better understand these different possible patterns of personality change across the semester vs. over the college years, as these trends may have important implications for supporting students through short-term challenges, knowing that maturity and growth are expected in the longer-term.

## Data Availability

The datasets presented in this study can be found in online repositories. The names of the repository/repositories and accession number(s) can be found below: https://osf.io/f2m6d/files/osfstorage.

## Footnotes

^1^Two hundred and eighty-two participated in Part 2, but two participants did not complete the Big Five Inventory at T2.

^2^Because the composite academic effort and social activity change variables contained items measured in hours per week, T1–T2 difference scores were first calculated for the unstandardized individual item pairs; the difference scores for each item pair in the composite were then standardized and averaged.

^3^Unexpectedly, for the academic effort, extracurricular/work, and social activity items measured in hours per week, a few participants provided unreasonably or impossibly high responses (e.g., estimates that exceeded the number of hours in a week). For items with extreme responses, we excluded responses that were greater than 3 standard deviations above the mean.

^4^Because over half of participants were first year students (many who had not yet declared a major), we did not analyze responses to the college major question. We also did not analyze the open-ended questions asking which psychology courses participants were currently enrolled in. In addition, because the three institutions measured course load differently (e.g., number of courses vs. credit hours), we did not analyze these variables.

^5^Standardized beta coefficients (βs) are reported for the LCSM and Cohen's *d* for the *t*-tests.

^6^We present exploratory two one-sided test (TOST) analyses testing for equivalence in extraversion and neuroticism at T1 and T2 in the Supplementary material.

^7^Exploratory cross-lag correlations between personality at T1 and experiences at T2, experiences at T1 and personality at T2, personality at T1 and changes in experiences, and experiences at T1 and changes in personality are presented in the Supplementary material.

^8^Although we preregistered plans to conduct robustness checks controlling for demographic variables, we determined that controlling for demographics did not make sense theoretically (especially age, which is relevant to the personality changes we sought to test). Instead of controlling for these variables, we tested for possible interactive effects with gender and age, which are more theoretically relevant and logical analyses to perform considering prior research indicating age and gender differences in personality and personality change (e.g., Roberts and Mroczek, 2008; Roberts et al., 2006; Soto et al., 2011; Weisberg et al., 2011). The analyses for gender only included men and women, given the limited number of participants who did not identify as a man or woman.

^9^The decline in overall social activity was observed in the LCSM but not in the *t*-tests.
