# Supplementary material for: Personality stability and change across the academic semester
Source: Front Psychol. 2025 Jun 27;16:1531794. doi: 10.3389/fpsyg.2025.1531794 (PMC12247851; doi:10.3389/fpsyg.2025.1531794)
Supplement: Supplementary file 1 [file Supplementary_file_1.pdf]

Supplementary Materials

Exploratory Equivalence Tests

We performed exploratory (i.e., post hoc) two one-sided test (TOST) analyses to test for equivalence in extraversion and neuroticism at T1 and T2 (i.e., statistically reject the presence of an effect based on the smallest effect size of interest). For the equivalence tests, we calculated an upper and lower bound (Δ_L, Δ_U) that represents the smallest effect size of interest, using d = .20, the effect size specified in the power analysis. First, we computed a confidence interval for d = .20 by multiplying d by the pooled standard deviation to obtain Δ and set Δ_L and Δ_U (Lakens, 2017). We then calculated the observed 90% confidence interval for the contrast and compared whether the 90% confidence interval fell within the upper and lower bounds for d = 0.20 (i.e., [Δ_L, Δ_U]). A 90% confidence interval falling within the upper and lower bound would allow us to significantly reject the alternative hypothesis of personality change, providing support for the null hypothesis of equivalence at T1 and T2, if the smallest effect size of interest is d = 0.20 (Lakens, 2017). A 90% confidence interval falling outside of these bounds would indicate that the study could not discriminate between the alternative hypothesis of personality change or null hypothesis of no change, possibly due to a lack of power. The TOST analyses showed that equivalence could not be determined at T1 and T2 for both neuroticism, 90% CI = [-.03, .28] vs. Δ_L, Δ_U = [-.15, .15], and extraversion, 90% CI = [-.19, .12] vs. Δ_L, Δ_U = [-.13, .13], suggesting the study could not distinguish between change or no change in these traits. Thus, further research is needed to conclusively determine whether neuroticism and extraversion show patterns of change across the semester or not.

Exploratory Cross-Lag Correlations

Table S1. Cross-Lag Correlations between Personality at T1 and Experiences at T2

	C	N	E	O	A
1. Subjective Well-Being					
Composite	.24*	-.52*	.31*	.04	.37*
Stressed	-.12	.39*	-.16*	-.02	-.16*
Anxious	-.14*	.45*	-.08	.004	-.20*
Sad	-.22*	.44*	-.18*	.04	-.30*
Happy	.13*	-.29*	.33*	.05	.34*
Life satisfaction	.26*	-.32*	.38*	.11	.35*
2. Social Support					
Composite	.20*	-.30*	.36*	.10	.30*
Socially supported	.20*	-.22*	.42*	.15*	.21*
Satisfied with relationships	.16*	-.23*	.32*	.04	.23*
Conflict in relationships	-.10	.26*	-.10	-.05	-.25*
3. Health Behaviors					
Composite	.28*	-.37*	.29*	-.02	.27*
Healthy eating	.32*	-.27*	.27*	-.02	.18*
Exercise	.24*	-.20*	.25*	-.04	.11
Trouble sleeping	-.12*	.24*	-.10	.004	-.27*
Tiredness	-.12*	.28*	-.20*	.003	-.12*
Sickness	-.09	.21*	-.13*	-.01	-.19*
4. Academic Attendance and Effort					
Composite (standardized)	-.01	.14*	.02	-.08	-.05
Time in class	.02	-.05	.01	-.05	.06
Time on coursework	.07	.08	.05	.02	-.01
Class absences	-.11	.21*	-.02	-.12*	-.15*
5. Extracurricular and Work Activities					
Composite time (standardized)	--	--	--	--	--
Time on extracurriculars	.11	-.11	.17*	.02	.09
Time working paid job/internship	.08	-.05	.05	-.001	-.02
Time working unpaid job/internship	-.14*	.02	.02	-.002	-.08
Time spent volunteering	.06	.02	.07	-.04	.06
Trying new activities	.06	-.06	.05	.08	.06
6. Social Activity					
Composite (standardized)	.01	.03	.24*	-.01	.02
Free time spent with friends	-.03	.03	.18*	-.03	-.02
Time attending social events	.05	.03	.20*	.02	.05

**Note*. $p < .05$.

Table S2. Cross-Lag Correlations between Experiences at T1 and Personality at T2

	C	N	E	O	A
1. Subjective Well-Being					
Composite	.18*	-.44*	.21*	.07	.18*
Stressed	-.13*	.33*	-.04	-.01	-.11
Anxious	-.10	.39*	-.07	-.01	-.05
Sad	-.05	.27*	-.08	.01	-.13*
Happy	.15*	-.28*	.32*	.16*	.18*
Life satisfaction	.20*	-.26*	.26*	.11	.20*
2. Social Support					
Composite	.27*	-.27*	.24*	.002	.23*
Socially supported	21*	-.18*	.32*	.12*	.21*
Satisfied with relationships	.20*	-.21*	.28*	-.03	.13*
Conflict in relationships	-.18*	.21*	.06	.09	-.17*
3. Health Behaviors					
Composite	.39*	-.46*	.24*	.06	.20*
Healthy eating	.36*	-.31*	.23*	.10	.12
Exercise	.23*	-.18*	.16*	.02	-.001
Trouble sleeping	-.13*	.27*	-.10	-.03	-.21*
Tiredness	-.24*	.39*	-.17*	-.04	-.18*
Sickness	-.26*	.28*	-.10	-.01	-.12
4. Academic Attendance and Effort					
Composite (standardized)	.01	.04	.02	.08	.10
Time in class	.07	-.10	.07	.01	.04
Time on coursework	.15*	.06	.01	.14*	.10
Class absences	-.19*	.11	-.04	.01	.04
5. Extracurricular and Work Activities					
Composite time (standardized)	--	--	--	--	--
Time on extracurriculars	.14*	-.05	.16*	.12	-.02
Time working paid job/internship	.11	-.03	.06	-.03	-.06
Time working unpaid job/internship	-.01	.01	.04	.08	-.04
Time spent volunteering	-.01	.01	.08	.14*	.01
Trying new activities	.11	-.09	.10	.15*	.05
6. Social Activity					
Composite (standardized)	.08	-.19*	.20*	-.03	.17*
Free time spent with friends	.06	-.13*	.14*	-.07	.16*
Time attending social events	.07	-.20*	.21*	.03	.14*

**Note*. $p < .05$.

Table S3. Cross-Lag Correlations between Personality at T1 and Changes in Experiences

	C	N	E	O	A
1. Subjective Well-Being					
Composite	-.04	.17*	-.06	<.001	.05
Stressed	.12	-.15*	-.01	-.06	.03
Anxious	-.003	-.16*	.10	-.09	-.09
Sad	-.07	-.09	-.01	-.01	-.03
Happy	-.07	.11	-.10	-.13*	-.003
Life satisfaction	-.02	.06	-.02	-.04	.05
2. Social Support					
Composite	-.04	.07	.001	.01	-.02
Socially supported	-.02	.07	-.01	-.04	-.02
Satisfied with relationships	-.04	.03	-.08	-.12*	.02
Conflict in relationships	-.22*	.37*	-.35*	-.16*	-.33*
3. Health Behaviors					
Composite	-.16*	.06	.002	-.03	-.02
Healthy eating	-.05	.02	<.001	-.06	-.01
Exercise	-.02	-.04	.09	.08	.04
Trouble sleeping	.09	.01	.04	.05	-.02
Tiredness	.11	-.15*	.02	.003	.08
Sickness	.14*	-.03	.02	.05	.02
4. Academic Attendance and Effort					
Composite (standardized)	-.05	.03	.01	-.11	-.08
Time in class	-.03	-.07	-.07	-.01	.02
Time on coursework	-.09	-.02	.08	-.12*	-.09
Class absences	.04	.13*	.01	-.08	-.08
5. Extracurricular and Work Activities					
Composite time (standardized)	--	--	--	--	--
Time on extracurriculars	-.02	-.07	.04	-.06	.11
Time working paid job/internship	-.06	-.04	-.01	-.05	.02
Time working unpaid job/internship	-.12*	.06	-.05	-.09	-.02
Time spent volunteering	.04	-.02	.06	-.07	.02
Trying new activities	-.11	.11	-.10	-.05	-.14*
6. Social Activity					
Composite (standardized)	-.01	.15*	-.09	.03	-.11
Free time spent with friends	-.07	.13*	-.07	.02	-.08
Time attending social events	.05	.11	-.08	.02	-.10

***Note*. $p < .05$.

Table S4. Cross-Lag Correlations between Experiences at T1 and Changes in Personality

	C	N	E	O	A
1. Subjective Well-Being					
Composite	-.12	.27*	-.20*	.03	-.12*
Stressed	.09	-.18*	.10	-.05	.04
Anxious	.04	-.22*	.14*	-.11	.05
Sad	.12*	-.30*	.11	-.04	.12*
Happy	-.08	.16*	-.19*	-.05	-.17*
Life satisfaction	-.08	.11	-.16*	-.05	-.07
2. Social Support					
Composite	.02	.15*	-.18*	-.11	-.09
Socially supported	-.01	.12*	-.15*	-.09	.01
Satisfied with relationships	.04	.10	-.16*	-.11	-.08
Conflict in relationships	-.002	-.12*	.09	.05	.14*
3. Health Behaviors					
Composite	-.01	-.02	-.08	.06	-.07
Healthy eating	.02	-.02	-.05	.07	-.05
Exercise	-.01	-.02	-.01	.12*	-.07
Trouble sleeping	.08	.03	.06	.02	.004
Tiredness	-.03	-.03	.06	-.04	-.02
Sickness	-.02	.01	.08	.06	.10
4. Academic Attendance and Effort					
Composite (standardized)	.03	-.08	.02	.05	.11
Time in class	.02	-.15*	-.03	.05	.01
Time on coursework	.03	-.03	.05	.002	.05
Class absences	.02	.02	.03	.06	.14*
5. Extracurricular and Work Activities					
Composite time (standardized)	--	--	--	--	--
Time on extracurriculars	-.01	.03	-.003	.01	-.07
Time working paid job/internship	-.01	.003	.01	-.07	-.05
Time working unpaid job/internship	.001	.07	-.05	-.06	.03
Time spent volunteering	-.05	.07	.01	.10	-.04
Trying new activities	-.09	.05	-.11	.01	-.18*
6. Social Activity					
Composite (standardized)	.07	-.07	-.14*	.001	.06
Free time spent with friends	.03	-.04	-.11*	-.03	.11
Time attending social events	.09	-.09	-.13*	.04	-.02

**Note*. $p < .05$.

Supplemental Correlational Analyses for T1 and T2 Experience Measures

Table S5. Subjective Well-Being Correlations at T1 and T2

	1	2	3	4	5	6	7	8	9	10	11
1. SWB, T1	--										
2. Stressed, T1	-.71*	--									
3. Anxious, T1	-.78*	.70*	--								
4. Sad, T1	-.74*	.42*	.50*	--							
5. Happy, T1	.63*	-.16*	-.24*	-.29*	--						
6. Life satisfaction, T1	.66*	-.16*	-.21*	-.35*	.62*	--					
7. SWB, T2	**.53***	-.39*	-.38*	-.35*	.39*	.34*	--				
8. Stressed, T2	-.40*	**.45***	.39*	.22*	-.16*	-.15*	-.74*	--			
9. Anxious, T2	-.44*	.47*	**.51***	.27*	-.16*	-.09	-.75*	.71*	--		
10. Sad, T2	-.38*	.26*	.25*	**.41***	-.20*	-.22*	-.79*	.49*	.52*	--	
11. Happy, T2	.32*	-.11	-.11	-.17*	**.45***	.32*	.68*	-.22*	-.26*	-.45*	--
12. Life satisfaction, T2	.38*	-.11	-.12*	-.20*	.47*	**.48***	.68*	-.28*	-.22*	-.41*	.59*

**Note*. $p < .05$. Bolded correlations highlight consistency between T1 and T2 measures.

Table S6. Social Support Correlations at T1 and T2

	1	2	3	4	5	6	7
1. Social support composite, T1	--						
2. Social support, T1	.76*	--					
3. Relationship satisfaction, T1	.84*	.61*	--				
4. Relationship conflict, T1	-.63*	-.11*	-.27*	--			
5. Social support composite, T2	**.45***	.38*	.41*	-.21*	--		
6. Social support, T2	.39*	**.45***	.41*	-.01	.79*	--	
7. Relationship satisfaction, T2	.38*	.33*	**.44***	-.07	.87*	.69*	--
8. Relationship conflict, T2	-.28*	-.12	-.12*	**.39***	-.68*	-.18*	-.35*

**Note*. *p* < .05. Bolded correlations indicate consistency between T1 and T2 social support measures.

Table S7. Health Behavior Correlations at T1 and T2

	1	2	3	4	5	6	7	8	9	10	11
1. Health Composite, T1	--										
2. Healthy Eating, T1	.61*	--									
3. Exercise, T1	.64*	.48*	--								
4. Trouble Sleeping, T1	-.60*	-.07	-.08	--							
5. Tired, T1	-.60*	-.18*	-.08	.40*	--						
6. Sick, T1	-.60*	-.17*	-.21*	.23*	.25*	--					
7. Health Composite, T2	**.67***	.47*	.36*	-.43*	-.43*	-.36*	--				
8. Healthy Eating, T2	.48*	**.61***	.36*	-.13*	-.22*	-.18*	.63*	--			
9. Exercise, T2	.51*	.44*	**.63***	-.11	-.14*	-.18*	.60*	.48*	--		
10. Trouble Sleeping, T2	-.42*	-.17*	-.06	**.50***	.33*	.26*	-.72*	-.28*	-.14*	--	
11. Tired, T2	-.38*	-.15*	-.11	.25*	**.48***	.19*	-.65*	-.19*	-.14*	.49*	--
12. Sick, T2	-.36*	-.15*	.004	.38*	.26*	**.34***	-.61*	-.12*	-.11	.38*	.34*

**Note*. $p < .05$. Bolded correlations indicate consistency between T1 and T2 health behavior measures.

Table S8. Academic Effort Correlations at T1 and T2

	1	2	3	4	5	6	7
1. Academic effort, T1	--						
2. Time in class, T1	.62*	--					
3. Time on coursework, T1	.72*	.30*	--				
4. Class absences, T1	-.46*	.18*	.01	--			
5. Academic effort, T2	**.27***	.21*	.22*	-.06	--		
6. Time in class, T2	.06	**.20***	-.002	.09	.60*	--	
7. Time on coursework, T2	.39*	.15*	**.53***	-.01	.71*	.24*	--
8. Class absences, T2	-.04	-.03	.13*	**.18***	-.47*	.19*	-.01

**Note*. $p < .05$. Bolded correlations indicate consistency between T1 and T2 academic effort measures.

Table S9. Extracurricular/Work Correlations at T1 and T2

	1	2	3	4	5	6	7	8	9
1. Time on extracurriculars, T1	--								
2. Time working paid job, T1	-.05	--							
3. Time working unpaid job, T1	.05	.04							
4. Time volunteering, T1	.18*	.03	.14*						
5. New activities, T1	.11*	-.15*	.003	.05					
6. Time on extracurriculars, T2	**.60***	-.11	-.05	.07	.05				
7. Time working paid job, T2	-.02	**.79***	.07	.06	-.16*	-.06			
8. Time working unpaid job, T2	-.07	-.04	**.20***	.10	-.05	.004	.04		
9. Time volunteering, T2	.08	.03	-.01	**.40***	.06	.12*	.06	.16*	
10. New activities, T2	.02	.06	-.04	.13*	**.17***	.06	-.02	-.01	.20*

**Note*. $p < .05$. Bolded correlations highlight consistency between T1 and T2 extracurricular/work activity measures.

Table S10. Social Activity at T1 and T2

	1	2	3	4	5
1. Social activity composite, T1	--				
2. Time with friends, T1	.86*	--			
3. Time attending social events, T1	.86*	.46*	--		
4. Social activity composite, T2	**.38***	.39*	.26*	--	
5. Time with friends, T2	.38*	**.44***	.21*	.80*	--
6. Time attending social events, T2	.23*	.18*	**.21***	.80*	.28*

**Note*. *p* < .05. Bolded correlations highlight consistency between T1 and T2 social activity measures.

Table S11. Latent Change Score Model Fit Statistics

Model	*CFI*	*SRMR*	*RMSEA*	*AIC*	*BIC*	*ChiSq*	*df*	*Params*
Extraversion	0.83	0.08	0.09	14137.2	14273.6	478.49	117	35
Agreeableness	0.9	0.06	0.05	15411.3	15563.3	279.51	150	39
Conscientiousness	0.91	0.07	0.05	15916.3	16068.3	312.54	150	39
Neuroticism	0.92	0.06	0.06	14620	14756.4	261.05	117	35
Openness	0.94	0.06	0.03	17439.2	17606.7	262.89	187	43
Subjective Well-Being	0.77	0.12	0.14	8815.06	8904.69	344.29	42	23
Social Support	0.98	0.05	0.05	5528.13	5586.59	24.43	12	15
Health Behaviors	0.97	0.05	0.06	5752.2	5810.58	25.19	12	15
Extracurricular	0.86	0.08	0.07	14101.3	14190.8	112.19	42	23
Social Activity	0.99	0.03	0.04	8091.81	8134.59	5.18	3	11
